# Modulation of asthma and allergy by addressing toll-like receptor 2

**DOI:** 10.1186/1745-6673-3-S1-S5

**Published:** 2008-02-27

**Authors:** Barbara Fuchs, Armin Braun

**Affiliations:** 1 Department of Immunology, Allergology and Immunotoxicology, Fraunhofer Institute of Toxicology and Experimental Medicine, Nikolai-Fuchs-Str. 1, 30625 Hannover, Germany

## Abstract

Toll-like receptors play an important role in innate and adaptive immunity and in balancing immune responses with tolerance. TLR2 is related to protection against allergies and allergic asthma by sensing pathogen associated patterns as lipoproteins and lipopeptides. A constant Th1 triggering is thought to prevent Th2 related disorders.

TLR2 is expressed on a variety of cells, both structural as well as immune cells. Importantly, TLR2 is also expressed on dendritic cells, which are thought to be one of the key players of initiating and maintaining immune responses. Therefore, TLR2 on dendritic cells is a good target for modulating immunity either to Th1 or Th2 responses, or induction of tolerance.

TLR2 agonists show high immunomodulatory and adjuvantic capacity. This makes TLR2 agonisation a promising approach for pharmaceutical intervention of allergic disorders.

## Introduction

Since a human homologue to drosophila toll-receptor had been firstly described [1], the family of TLR increased in members. Furthermore, knowledge broadens about TLR, their role in innate and adaptive immunity and their implication in balancing immune responses with tolerance. One possible mechanism herein is suppression of CD4+CD25+ regulatory T cells, allowing the host to develop an adequate adaptive immune response against microbacteria [2].

Formulation of the hygiene hypothesis pointed out an inverse association of microbial load and Th2 disorders [3,4]. Additionally, genetic variations in TLR2, but not in TLR4 [5], seem to sign responsible for an observed protection of farmers' children from allergy and asthma [6]. These protective actions seem to be of special importance to start already during pregnancy, when prenatal exposure to farm stables upregulates TLR expression of neonatal cells [7].

On the other hand, smoking during pregnancy attenuates TLR-mediated immune responses, possibly increasing the risk for the offspring to develop allergies and asthma [8].

TLR2 is expressed on a variety of cells, both structural as well as immune cells, in humans and rodents as there are neutrophils [9], small airway epithelial cells as well as airway smooth muscle cells [10,11], tracheal muscle layer [12], monocytes [13], macrophages [14], glial cells [15], murine bone-marrow derived mast cells [16], and B cells [17,18]. Its expression is inducible by TNF-α and IFN-γ.

Very importantly, TLR2 is also expressed on DCs, which are thought to be one of the key players of initiating and maintaining immune responses, and therefore are a good target cell for modulating immunity either to Th1 or Th2 responses, or induction of tolerance [19-21].

TLR2 in general senses lipopeptides and lipoproteins, whereby different heterodimers recognise different structures: diacylated lipopeptides, e.g. MALP-2 [22], require TLR2/6 [9,23], whereas triacylated lipopeptides, e.g. Pam_3_CysSK_4_, are recognised by TLR2/1 [9] and lipoproteins by TLR2/4 [24].

Although effects of TLR2 agonisation are dependent from age of the experimental animal, such a correlation is not observed in humans so far [14,25].

Nevertheless, animal models remain a useful tool to investigate preventive or therapeutic effects related to TLR2.

## Effects of TLR2 agonisation

Administration of MALP-2 into the airways attracts neutrophils to the bronchoalveolar space within 24 h. Two to three days after instillation, macrophages become more prominent. On macrophages, TLR2 agonists show clear activating effects [26]. After 72 h, lymphocytes, although less in number, reach their maximum contribution to cellularity of BALF. These effects revealed after 10 d [27]. Furthermore, changes in lung histology occur after MALP-2 aerosol administration, where the area of bronchus-associated lymphoid tissue is increased. The functional relevance of this finding remains to be investigated [28,29].

## Immunostimulation in allergy and allergic asthma

TLR2 agonisation bears the potency to both inhibit and promote development of immune responses and is therefore manifold in its implementation.

Mycoplasma infections prevent asthma, an effect which is partly dependent on the TLR2-IFN-γ -pathway [30]. This finding lead to the development of small Mycoplasma-derived compounds for potential pharmacological intervention of allergic diseases. A modulation of an already existing allergy could be achieved by using such Mycoplasma-derived compounds, as for example MALP-2. Intratracheal treatment with this TLR2/6 agonist in combination with the Th1-cytokine IFN-γ clearly reduced AHR, eosinophilia and Th2 cytokines in BALF; however, neutrophils and IL-12p70 were induced [31]. Likewise, treatment with a synthetic TLR2/1 ligand reduced total cell as well as eosinophil counts in the BALF, IL-4 and IL-5 levels as well as AHR. These reductions were independent from IL-10 and TGF-β [32], implicating rather a shift to a Th1 reaction than an induction of tolerance to be responsible for these observations. Additionally, TLR2/4 agonisation during allergen challenge in sensitised mice prevented allergic asthma. On DCs, IL-12 and TNF-α were induced, which by itself induces IFN-γ production of T lymphocytes. As a result, eosinophils, IL-4 and IL-13 were reduced, while neutrophil counts and IFN-γ were elevated, and no increased activation of Th1-lymphocytes could be detected [24]. However, also the contrary effect could be observed: TLR2/1 agonisation aggravated allergic asthma when administered during the initial phase of the immune reaction. The type of TLR stimulation during this early phase seems to be a determinant for the polarisation of the adaptive immune response [33]. When TLR2 ligands were administered during the efferent phase in a murine model of allergic conjunctivitis, the infiltration of eosinophils was suppressed, but rather by inducing a CD4+ cells apoptosis than by inducing a Th1 response [34]. Investigations in an in vitro model of allergy demonstrated an induction of TNF-α and IL-10 synthesis, but not IL-12, when blood derived DCs were stimulated with MALP-2 [35]. All these examples demonstrate the various implementations of TLR2 agonisation, either for shifting Th2 towards Th1, aggravating Th2 or induction of tolerance.

Its high immunomodulatory capacity as an adjuvant is further emphasised in experimental vaccination against HIV and measles [36-39]. This makes TLR2 agonisation a promising approach for pharmaceutical intervention.

TLR2 expression and function is influenced by administration of steroids, e.g. dexamethasone. On human airway smooth muscle cells, upregulation by cytokines as IFN-γ and TNF-α is potentiated; however, dexamethasone alone suppresses receptor expression [10]. This might be an explanation of infectious exacerbations occurring in steroid treated asthma, in contrast to viral exacerbations mediated via TLR3 [11].

## Conclusion

TLR2 is an important receptor in innate and adaptive immunity and related to protection against allergic disorders in humans. Due to their high immunomodulatory and adjuvantic capacity, TLR2 agonists bear manifold implications. Therefore, TLR2 agonists may provide potent new strategies either for prevention or treatment of allergies and allergic asthma.

## Competing interests

The authors declare that they have no competing interests.

## Authors' contributions

BF drafted the manuscript. AB discussed and corrected the manuscript.

All authors read and approved the final manuscript.
